# Brain morphometric abnormalities in boys with attention‐deficit/hyperactivity disorder revealed by sulcal pits‐based analyses

**DOI:** 10.1111/cns.13445

**Published:** 2020-08-06

**Authors:** Xin‐Wei Li, Yu‐Hao Jiang, Wei Wang, Xiao‐Xue Liu, Zhang‐Yong Li

**Affiliations:** ^1^ Chongqing Engineering Research Center of Medical Electronics and Information Technology Chongqing University of Posts and Telecommunications Chongqing China; ^2^ Chongqing Engineering Laboratory of Digital Medical Equipment and Systems Chongqing University of Posts and Telecommunications Chongqing China

**Keywords:** attention‐deficit/hyperactivity disorder, cortical morphology, magnetic resonance imaging, neurodevelopment, sulcal pits

## Abstract

**Aim:**

Attention‐deficit/hyperactivity disorder (ADHD) is a common neurodevelopmental disorder associated with widespread brain morphological abnormalities. Here, we utilized a sulcal pits‐based method to provide new insight into the atypical cortical folding morphology in ADHD.

**Methods:**

Sulcal pits, the locally deepest points in each fold, were first extracted from magnetic resonance imaging data of 183 boys with ADHD (10.62 ± 1.96 years) and 167 age‐ and gender‐matched typically developing controls (10.70 ± 1.73 years). Then, the geometrical properties of sulcal pits were statistically compared between ADHD and controls.

**Results:**

Our results demonstrated that the number of sulcal pits was reduced and confined to the superficial secondary sulci in the ADHD group relative to controls (*P* < .05). We also found that ADHD boys were associated with significantly increased pit depth in the left superior frontal junction, circular insular sulcus, right inferior frontal junction, and bilateral cingulate sulcus, as well as significantly decreased pit depth in the bilateral orbital sulcus (*P* < .05, corrected).

**Conclusion:**

The experimental findings reveal atypical sulcal anatomy in boys with ADHD and support the feasibility of sulcal pits as anatomic landmarks for disease diagnosis.

## Graphical abstract

Sulcal pits analysis revealed decreased pits number in superficial secondary sulci and abnormal pit depth in multiple brain regions in boys with ADHD, providing new insights into this disease.

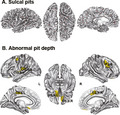

## 1 INTRODUCTION

Attention‐deficit/hyperactivity disorder (ADHD) is a common neurodevelopmental disorder, primarily characterized by difficulties in inattention, hyperactivity, and/or impulsivity.^1^ Although the underlying neurobiological substrate of ADHD is not fully understood, abundant neuroimaging studies have revealed widespread brain morphological abnormalities in ADHD. For example, many studies applying voxel‐based morphometry analysis have found altered gray matter volumes in several brain regions, such as the frontal, parietal cortex, and subcortical regions.^2^, ^3^, ^4^ Besides, some studies using surface‐based morphological methods have confirmed that ADHD is accompanied by thinner cortical thickness and lower cortical surface area.^5^, ^6^, ^7^ Moreover, a recent study focused on specific sulcus have reported regional shape abnormalities of the central sulcus in children with ADHD.^8^

In addition to the above morphological analysis methods, sulcal pits have received increasing attention in the past few years, which can provide valuable insight into the development of cortical folding.^9^ Sulcal pits are defined as the locally deepest points of primary sulci in the cerebral cortex, which are thought to be the first to develop embryologically and show few interindividual variabilities.^10^ Im et al have demonstrated that sulcal pits can be reliably extracted over different scan sessions, scanners, and surface extraction tools.^11^ Besides, sulcal pits are believed to be under tight genetic control, which is supported by the heritability estimates of the pit depth in a large human pedigree cohort.^12^ Studies have also shown that sulcal pits are closely related to the functional organization of the cortex.^13^, ^14^, ^15^

Moreover, a few studies have already demonstrated the feasibility of using sulcal pits as anatomic landmarks to distinguish normal controls from diseased ones. For instance, Brun et al found a reduced pit depth in the left ascending ramus of the Sylvian fissure in children with autistic disorder relative to controls.^16^ Using a pit‐based graph matching technique, Im et al revealed an atypical sulcal pattern in the left parieto‐temporal and occipito‐temporal regions in children with developmental dyslexia,^17^ and Ortinau et al reported an early emerging atypical sulcal pattern in fetuses with congenital heart disease.^18^ These studies showed that sulcal pits are suitable to uncover atypical cortical folding in the diseased brain.

However, although widespread morphological abnormalities in ADHD have been reported, the effect of this disease on the sulcus has not been fully elucidated. Therefore, the main aim of this study was to use sulcal pits to investigate the atypical cortical folding in ADHD, which may provide new insights into this disease. We first extracted sulcal pits in native space from magnetic resonance imaging (MRI) data of 183 boys with ADHD and 167 age‐ and gender‐matched typically developing controls (TDC) using an adaptive watershed algorithm. Next, we parcellated the white matter surface into several regions based on the group spatial distribution of sulcal pits. Then, the number of pits in deep/shallow folds and pit depth in each area was compared between groups. Finally, we investigated whether the hemispheric asymmetry of sulcal pits could distinguish ADHD patients from normal controls.

## 2 MATERIALS AND METHODS

### 2.1 Subjects

The MRI data utilized in this study were obtained from the Neuro Bureau ADHD‐200 Preprocessed repository.^19^ The ADHD‐200 database consists of 973 individuals (ages: 7‐21 years old) collected from eight contributing sites, including 585 typically developing controls (TDC), 362 patients with ADHD, and 26 participants with diagnosis unavailable. A subsample was selected according to the following criterions: (a) Only sites with both diagnostic groups (ADHD and TDC) were retained to minimize variability across imaging sites; (b) subjects failed quality control of anatomic images were excluded; (c) female subjects were excluded to remove the confounding effect of gender; (d) only subjects between the ages of 7 and 14 were included to minimize potential developmental effects beyond a linear age effect that we considered in our statistical models in this study; (e) only right‐handedness subjects were included; (f) subjects with IQ lower than 80 or missing IQ were excluded; and (g) subjects diagnosed as ADHD‐hyperactive/impulsive subtype were excluded due to their under‐represented in the database.

Finally, a sample of 350 male right‐handed subjects (N* = *350, 183 ADHD and 167 TDC, ages: 7‐14 years old) was included in the study, with sample characteristics presented in Table 1. These subjects were from 4 sites, including 139 subjects from Peking University, 48 from Kennedy Krieger Institute, 107 from New York University, and 56 from Oregon Health & Science University. To facilitate the reproducibility of this study by others, the exact list of subjects is available in the Appendix S1 (see Tables S1 and S2). The detailed MRI scan parameters of these four sites can be found at the ADHD‐200 website http://fcon_1000.projects.nitrc.org/indi/adhd200/.

**Table 1:** Demographic of the subjects in the study

	ADHD (n = 183)	TDC (n = 167)	*P*‐value
Age (y)	10.6 ± 1.96	10.7 ± 1.73	0.70
ADHD type	119 combined, 64 inattentive	N/A	N/A
Sex	All males	All males	Matched
Handedness	All right‐handed	All right‐handed	Matched
FIQ	106.8 ± 13.7	116.0 ± 13.4	0.001

Abbreviations: ADHD, attention‐deficit/hyperactivity disorder; FIQ, full intelligence quotient; TDC, typically developing children.

All procedures performed were in accordance with the ethical standards of the institutional and/or national research committee and with the 1964 Helsinki declaration and its later amendments or comparable ethical standards. Informed consent was obtained from all individual participants included in the study.

### 2.2 Image preprocessing

The structural T1‐weighted MRI data were processed using the routine Freesurfer v5.3.0 pipeline.^20^ Briefly, the processing includes motion correction, Talairach transformation, intensity normalization, skull stripping, gray‐white matter segmentation, cortical surface reconstruction, fix topology, and cortical surface registration to a stereotaxic atlas. In the Freesurfer outputs, the white matter surfaces and registration sphere files were used for further analysis.

### 2.3 Sulcal pits extraction

**FIGURE 1:**
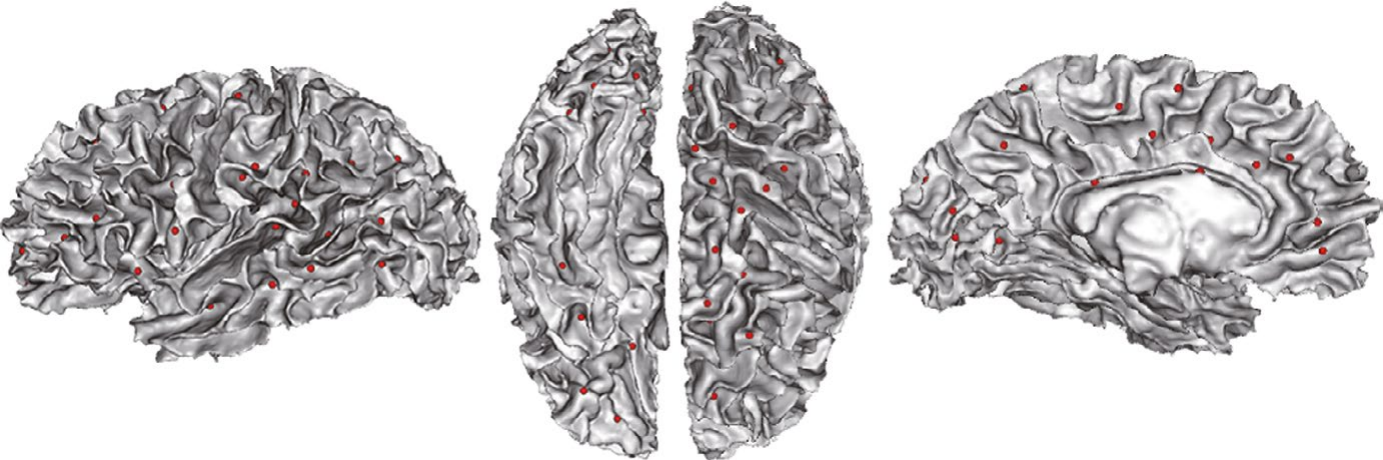
Sulcal pits extraction. The maps show the sulcal pits (red spheres) extracted from an individual's left hemisphere in native space

In this study, we adopted the method proposed by Auzias et al^13^ to extract deep sulcal pits from the white matter surfaces in native space using the BrainVISA process “Sulcal Pits Extraction” (http://brainvisa.info, version 4.6.1 built on Linux Ubuntu 16.04). This method was not influenced by individual brain size.^13^ First, the depth potential function (DPF) was used to estimate the depth of each vertex on the white matter surface to obtain a depth map.^21^ The DPF is a measure of the depth of the folds that combines curvature and convexity information. Then, a filtered watershed algorithm was applied to the depth map to divide the surface into multiple sulcal basins and locate the deepest point of each basin (ie, sulcal pit, see Figure 1). During the watershed flooding process, the sulcal basins were merged according to the ridge height (ie, the height difference between the ridge point and the shallowest pit, R), the basin area (A), and the geodesic distance between two pits (D). Specifically, during the flooding, two basins were merged if R < ThR and D < ThD, and after the flooding, the small basin (A < ThA) was merged with the neighbor which shares the longest boundary. The corresponding thresholds were first set to the default values (ThR = 1.5, ThD = 20, ThA = 50), which were obtained from a population of healthy adults.^13^ Then, the ThD and ThA were normalized according to ThD = ThD * FL/G(FL) and ThA = ThA * SA/G(SA), where FL and SA represent the mesh Fiedler length (a geodesic distance related to the overall shape of an object) and surface area for each subject, and G(·) represents the corresponding group average values. The group parameters G(·) were calculated from all individuals, where G(FL) was set to 231.8 for the left and 232.9 for the right, and G(SA) was set to 91 616 for the left and 91 980 for the right.

### 2.4 Group clusters definition

**FIGURE 2:**
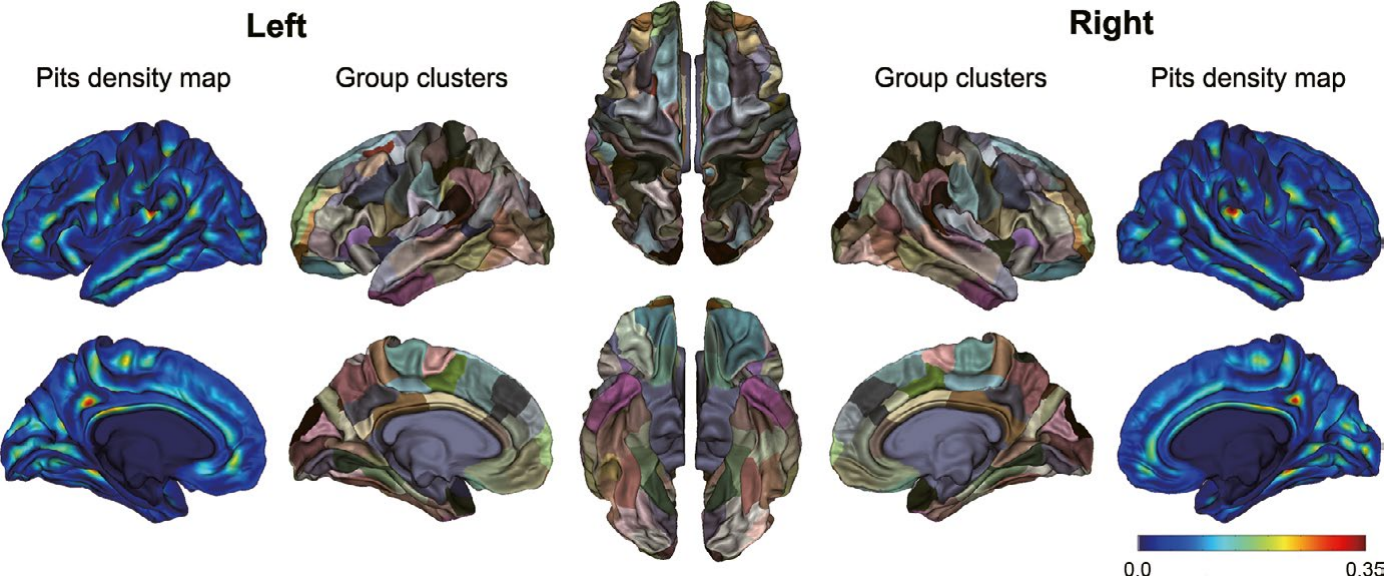
Final map of the group cluster regions and the density of sulcal pits. The group clusters were obtained by using the watershed algorithm on the pits density map

To compare the differences in sulcal pits between groups, we computed the group‐level clusters of pits as presented in Auzias et al^13^ We used all subjects to build the cluster map so that each diagnostic group could contribute equally to the final parcellation. First, the sulcal pits textures were smoothed using a full‐width half‐maximum (FWHM) of 5 mm, maintaining the peak value at one. This was done by solving the heat equation 60 times iteratively with the BrainVISA process “Pits Texture Smoothing.” Second, the smoothed pits' textures of both hemispheres were projected to the corresponding hemisphere of the Freesurfer fsaverage template. Third, all the pits textures were averaged across individuals to get a group density map showing the probability of pits at each location (Figure 2). Finally, the group density map was divided into several clusters (106 clusters for left and 99 clusters for right, see Figure 2) using a watershed algorithm, which was implemented by the BrainVISA process “Group Watershed.” The group watershed parameters were demonstrated to have a limited impact on the results^13^; thus, we used the default parameters (gThR = 2, gThD = 15, gThA = 100) in this study.

We also generated a symmetric group‐level clusters of pits for asymmetric analyses. The procedure was similar to the above, with the following modifications. First, we performed individual spherical interhemispheric registration to obtain the anatomic correspondences between the left and right cortical surfaces, as introduced in Greve et al^22^ Specifically, the right white matter surface was first transformed to the left side of the same person and then registered to the left side of the unbiased symmetric Freesurfer template (fsaverage_sym), while the left white matter surface was directly registered to the left side of the template. Second, all individual smoothed pits' textures of both hemispheres were projected to the left of the fsaverage_sym template and averaged. Then, the resulting density map was divided into several clusters (N = 104, see Figure 5A) as previously described.

### 2.5 Pits number analyses

**FIGURE 3:**
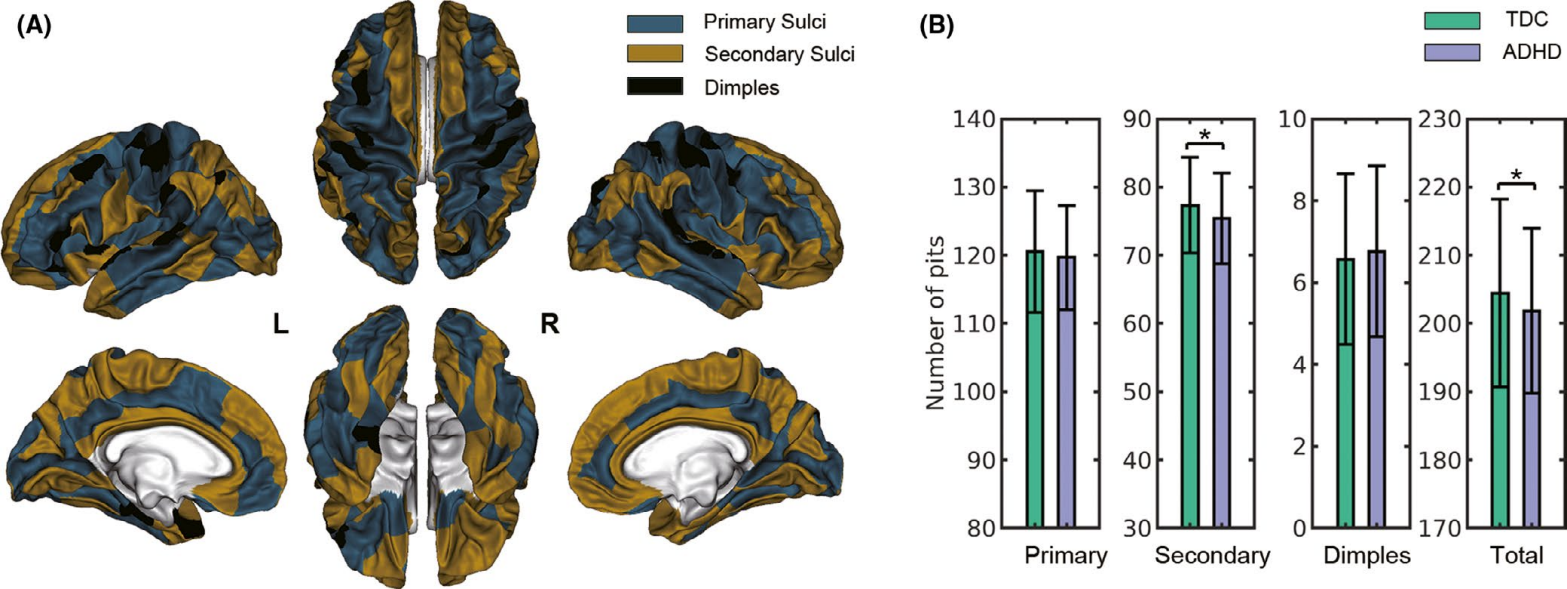
Results for the analyses of pits number. A, Division of cortical folds into primary sulci, secondary sulci and dimples based on depth. B, Bar charts depicting group differences of the pits numbers. L, left; R, right; TDC, typically developing children; ADHD, attention‐deficit/hyperactivity disorder; DPF, depth potential function; *P < .05

As the evolution processes of the deep primary sulci and superficial sulci are different,^23^ it is interesting to study the effect of ADHD on the number of sulcal pits within different sulcal classes separately. To do so, we first calculated average DPF of all subjects for each cluster and then classify the cortical folds using a k‐means clustering method (Replicates = 100). We first tested *k* = 2 but found that the primary sulci and superficial sulci could not be well distinguished. Then, we tested *k* = 3 and it worked out well. The resulting three classes of basins including the deepest basins identified as primary sulci, the shallow basins identified as secondary sulci, and the shallowest basins identified as “dimples” (see Figure 3A). Analyzing the three classes of sulci can also be found in Kruggel, 2018.^24^ Next, the number of sulcal pits across hemispheres in the primary sulci, secondary sulci, dimples, and the entire cortical folds was counted and compared between groups.

### 2.6 Pits DPF analyses

Next, we explored the effect of ADHD on sulcal pits at a finer scale, that is, pit depth in each cluster. For each group cluster, we measured the DPF of the pit within the cluster. If the cluster contains more than one pit, the DPF of the deepest pit was used. The left and right hemispheres were analyzed separately. For asymmetric analyses, we first examined within‐group hemispheric asymmetry in pit depth for both TDC and ADHD groups using the symmetric parcellations. Then, we analyzed the between‐group differences of pit depth using the asymmetry index (AI, also known as laterality index^22^) according to the formula: AI = (L−R)/(L + R), where L and R reflect the DPF of the (deepest) pit in the left or right hemisphere, respectively. The AI index ranges from −1 to +1, where −1 indicating completely right lateralized and +1 indicating completely left lateralized.

### 2.7 Statistical analyses

Statistical analyses were implemented in Python 2.7 using statsmodels 0.9. All statistical tests were two‐tailed. Linear regression models were used to explore the between‐group differences of the pits number in the primary sulci, secondary sulci, dimples, and the entire cortical folds, with the group as a dummy variable and age as a covariate. We also studied the linear effect of age on the pits number using simple regressions. The significance threshold was set at *P* < .05. Moreover, we applied linear regression models to examine between‐group differences in pit depth for each asymmetric cluster and the AI value of pit depth for each symmetric cluster, controlling for the age of the participants. Considering that the measurements of the left and right hemispheres are related, we used repeated‐measures ANOVA to test the differences in pits depth between the hemispheres for each group. For all cluster‐based statistical testing, *P*‐values <.05 were considered to be statistically significant, and the false discovery rate (FDR) procedure was used for multiple comparisons corrections (number of tests/clusters = 205 or 104 for the corresponding parcellation). It is worth noting that although ADHD patients have typically lower IQ than normal controls,^25^ we did not use IQ as a covariate for statistical analyses, as suggested by Dennis et al^26^ In addition, comparison of ADHD subtypes revealed no significant differences in pits number or depth. Thus, we did not treat the subtype as a covariate in the statistical analyses.

## 3 RESULTS

### 3.1 Pits number

Group comparisons of the number of sulcal pits are shown in Figure 3B and Table S3. At the brain level, we found a significant decrease in the total number of pits in the ADHD group compared to the TDC group (*P* = .02). When analyzing the deep early developing sulci, shallow later‐developing secondary sulci, and shallowest dimples separately, we observed that the number of pits in the secondary sulci was significantly reduced in ADHD patients compared with the control group (*P* = .01), but the number of pits in the deep primary sulci and dimples was not significantly different between groups (*P > *.05). In addition, we found that age had a significant linear effect on the pits number for all sulcal classes (*P* < .05).

### 3.2 Pits DPF

**FIGURE 4:**
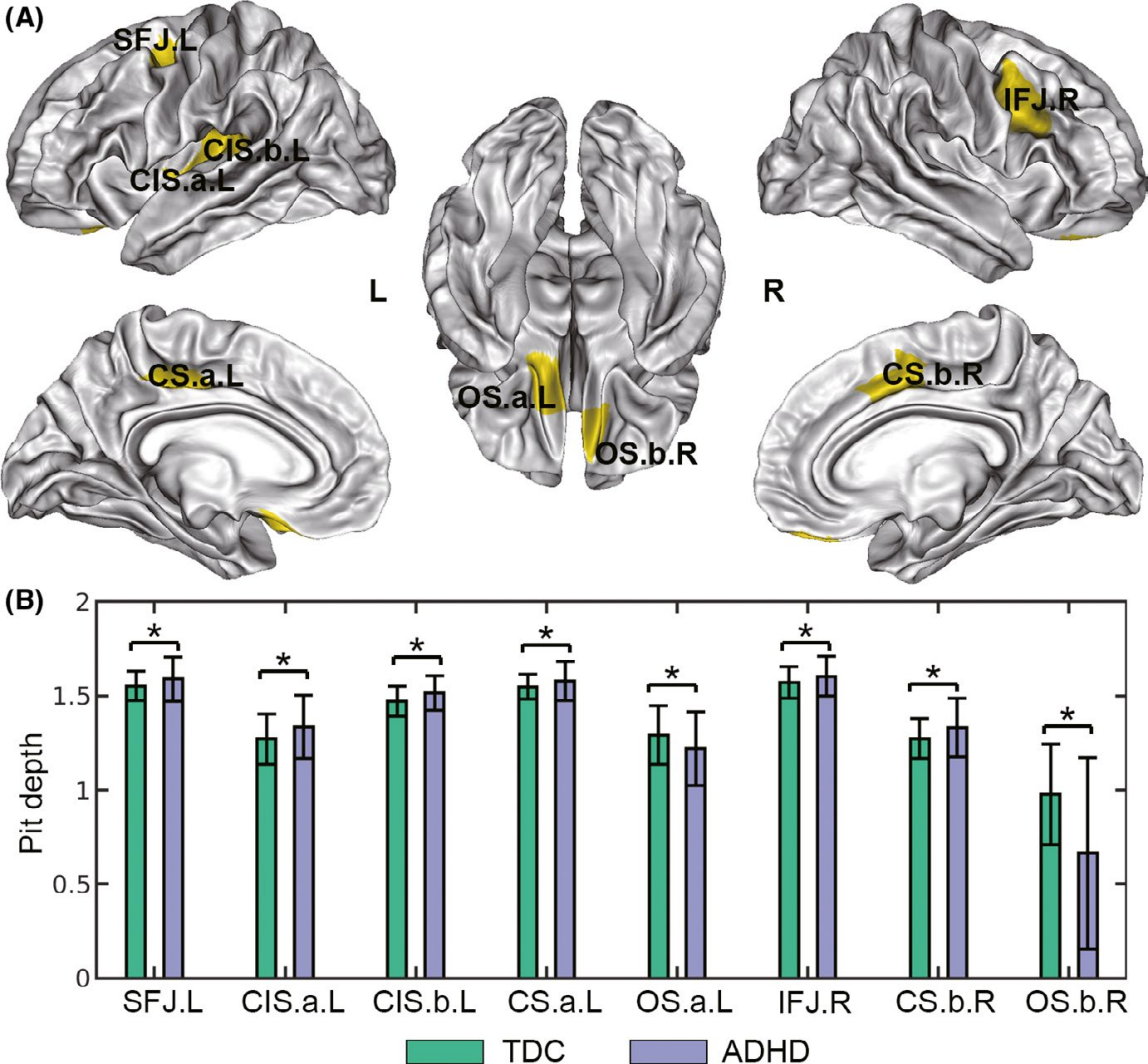
Results for the analyses of sulcal pit depth. A, 3D distribution of the brain regions with significant between‐group differences in sulcal pit depth at *P* < .05 (FDR corrected). B, Bar charts of the pit depth in TDC and ADHD groups for those significant regions. SFJ, superior frontal junction; CIS, circular insular sulcus; CS, cingulate sulcus; OS, orbital sulcus; IFJ, inferior frontal junction; A and B represent different regions of a sulcus. L, left; R, right; TDC, typically developing children; ADHD, attention‐deficit/hyperactivity disorder; *P < .05, FDR corrected

The 3D distribution of the aberrant clusters in pit depth (*P* < .05, FDR corrected) is shown in yellow in Figure 4A and the bar charts in Figure 4B provided additional statistical information. Compared with the TDC group, six clusters with increased pit depth were identified in the ADHD group, including left superior frontal junction (SFJ, at the junction between precentral sulcus and superior frontal sulcus, *P* = 8.56e‐4), circular insular sulcus (CIS, Region a: *P* = 9.41e‐6 and Region b: *P* = 9.80e‐4), right inferior frontal junction (IFJ, at the junction between precentral sulcus and inferior frontal sulcus, *P* = 7.27e‐4), and bilateral cingulate sulcus (CS, Left: *P* = 9.80e‐4, Right: 1.54e‐4), see Table S4. Moreover, the ADHD group had significantly shallower pit depth in bilateral olfactory sulcus (OS, left: *P* = 4.90e‐4 and right: *P* = 2.73e‐5). Nomenclature of the clusters mainly refers to the existing papers.^12^, ^14^, ^15^

### 3.3 Hemispheric asymmetry in pit depth

**FIGURE 5:**
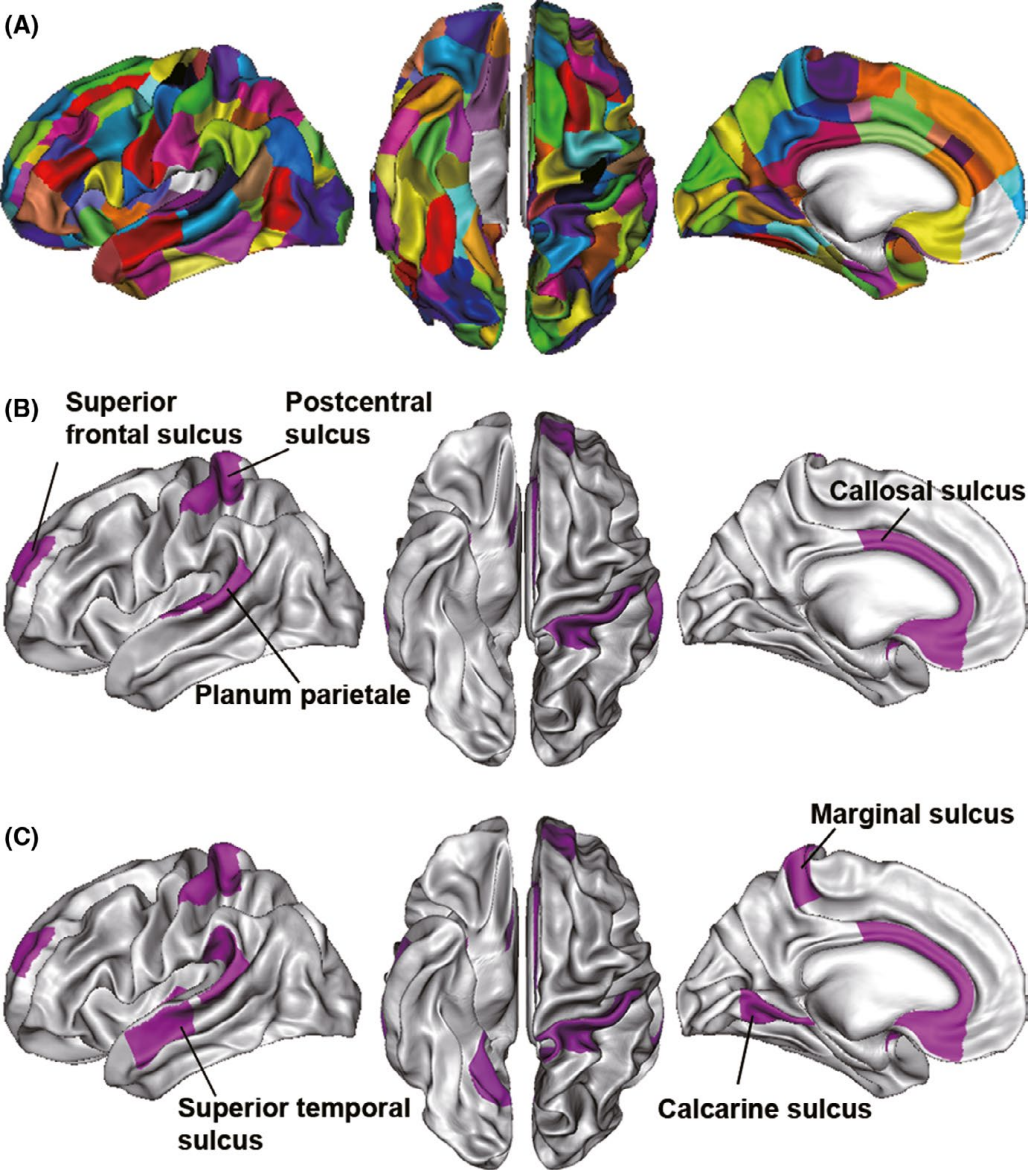
Within‐group hemisphere asymmetry in pit depth. A, show a map of symmetric group clusters. B and C, show a 3D distribution of the brain regions with significant asymmetry in typically developing children and attention‐deficit/hyperactivity disorder, respectively (*P* < .05, FDR corrected)

The within‐group asymmetries in pit depth for the TDC and ADHD groups are illustrated in Figure 5. Both groups showed significant asymmetry in planum parietale, callosal sulcus, postcentral sulcus, and superior frontal area in the pit depth analysis. Moreover, the ADHD group showed more asymmetric regions, including superior temporal sulcus, marginal sulcus, and calcarine sulcus. However, no significant differences in the AI index of the pit depth between the TDC and ADHD group were found after correction for multiple comparisons (*P* < .05, FDR corrected).

## 4 DISCUSSION

In this study, we described the first sulcal pits analysis of boys with ADHD and matched controls. Our findings indicated that ADHD boys have a reduced number of sulcal pits compared with TDC boys. We further observed that the difference only occurred in the superficial later‐developing folds but not in the deep early developing folds. Additionally, we found that ADHD subjects accompanied by increased pit depth in several regions, including left SFJ and CIS, right IFJ, and bilateral CS. Meanwhile, reduced pit depth of the bilateral OS was observed in the ADHD group relative to controls.

First of all, we found that the number of pits was reduced and confined to the superficial secondary sulci in the ADHD group. These results can also be observed using a different division technique of deep/superficial clusters (see Appendix S1 and Figure S1), indicating good robustness of these results. Since sulcal pits reflect the maturation of cortical folding, and the superficial folds are later‐developing, our results may provide support for the maturational delay model of ADHD. In support of this model, it has been shown that the cortical thickness and surface area of ADHD subjects peaked 2‐3 years later than controls, possibly due to global perturbation in the mechanisms that affecting brain maturation.^27^, ^28^ Besides, several previous studies of brain networks have found that ADHD patients associated with more centralized network structures, suggesting a developmental delay in ADHD.^29^, ^30^ However, the relationship between pits anomalies and cortical gyrification process needs to be further confirmed by longitudinal designs.

When investigated at a finer scale, we observed abnormal pit depth in multiple clusters in ADHD, which may indicate important disorganization of the relevant brain regions. Sulcal pit depth is associated with the gyrification process and can serve to describe the morphology of cortical folding.^9^ Their abnormalities may be associated with changes in the cortical thickness and volume. Vandekar et al reported that increased myelination plays an important role in the regional associations between sulcal depth and cortical thickness during development.^31^ In addition, previous studies have demonstrated that the relationship between sulcal depth and cortical thickness can be altered by brain disease. For example, Tosun et al found that these two indexes were significantly negatively correlated in the medial fronto‐orbital region in the TDC group and positively correlated in the precentral and postcentral regions in the focal epilepsy group.^32^ Besides, several studies have shown that sulcal pits can be used as anatomic landmarks that are closely related to brain function.^9^ Differences in pit depth may lead to functional abnormalities in ADHD, which remains to be further proved experimentally.

In ADHD, significantly deeper pit depth was found in the left SFJ regions. The SFJ is often called the frontal eye field, which has been shown to play a central role in modulating processing in visual cortex for spatial attention.^33^ Thus, structural changes to it may underpin attention problems in patients with ADHD. In fact, the SFJ has previously been identified as one of the main loci of structural abnormalities in ADHD.^34^, ^35^ Furthermore, the SFJ is a key node of the dorsal attentional network, which supports top‐down attentional control and is thought to be associated with the etiology of in ADHD.^36^ Previous neuroimaging studies have shown that the dorsal attentional network of ADHD patients is abnormally activated during executive and response inhibition tasks,^37^ and its laminar cortical thickness is relatively thinner than controls.^6^

Additionally, increased pit depth was found in the left CIS region in ADHD boys. According to Lopez‐Larson et al,^38^ the CIS section found in this study belongs to the posterior insula. We speculate that the involvement of posterior insula in ADHD may be associated with its role in sensorimotor processing.^39^, ^40^ While ADHD is typically characterized by inattention, hyperactivity, and impulsivity, this disorder is also associated with problems in sensorimotor function.^41^, ^42^ Indeed, a previous study has reported that the insula showed less activity in ADHD adults during sensorimotor timing tasks.^43^ However, many studies have suggested that the anterior insula is more involved in ADHD than the posterior insula.^38^, ^44^ The role of insular substructures in the pathological mechanisms of ADHD deserves further investigation.

Our results also revealed a deeper pit depth in the right IFJ in the ADHD group relative to the TDC group. Decreased gray matter volumes and thinning cortical thickness in this region of ADHD patients were observed.^45^, ^46^ Furthermore, Kelly et al detected weaker intrinsic functional connectivity within the right IFJ region in ADHD relative to controls.^47^ Moreover, Schilling et al reported the cortical thickness of the IFJ was significantly inversely correlated with impulsiveness, which is a central symptom in ADHD.^48^ Given the evidence that the IFJ region plays an important role in cognitive control,^49^, ^50^ our results may account for related deficits in ADHD patients.

We also observed deeper pit depth of the bilateral CS in ADHD boys compared to controls. The observed CS regions were located at the posterior part of the cingulate cortex, which is often found abnormal in ADHD. For example, using magnetoencephalography and functional MRI, Sudre et al confirmed that the posterior cingulate cortex was among the most atypical connectivity regions.^51^ Additionally, a voxel‐based morphometric study showed a reduction in posterior cingulate volume in ADHD.^3^ Moreover, there is increasing evidence that the posterior CS is involved in visual self‐motion processing.^52^ Further, a recent study of anatomic and functional connectivity suggests that CS (visual area) provides a link between perception and action to guide locomotion.^53^ Thus, the abnormalities of sulcal pits in the CS region may associate with ADHD deficits in motor function.

By contrast, the bilateral OS was the only region in which the ADHD group showed shallower pit depth. The OS region is one of the earliest developed brain regions and can be identified around 16 weeks of gestation.^54^ A significant relationship between its depth and olfactory function in healthy subjects was reported.^55^ Besides, shallower depth of the OS has been found in a variety of psychiatric disorders, indicating its potential as a pathognomonic marker for abnormal cortical development.^56^, ^57^ Our findings here provide new evidence that ADHD boys exhibit morphologic changes of the olfactory sulcus, which may account for deficits in olfactory function observed in ADHD.^58^, ^59^

Some important caveats should be considered in our study. First, the ADHD sample in this study was heterogeneous with two different subtypes, including about a third of the inattentive type and two‐thirds of the combined type. Future study can use a larger sample to compare abnormalities of sulcal pits between different ADHD subtypes. Second, only boys were included in our sample, so additional research is needed to determine whether our results could be generalized to girls with ADHD. Third, the unmatched IQ scores between the ADHD and TDC groups could be considered as bias, in particular concerning a previous study that suggests intelligence can affect the presence of sulcal pits.^60^ However, literature has shown that intelligence tests are affected by attention‐deficit, and ADHD patient scores are generally lower than controls.^25^ Thus, as suggested by Dennis et al,^26^ we did not match subjects with and without ADHD on IQ and did not treat IQ as a confounding covariate in statistical analysis. Furthermore, we only selected subjects with an IQ ≥ 80 to avoid the effect of low intellectual functioning on the results. However, this IQ cutoff may limit the broad applicability of our findings. Future work should recruit a larger sample size to explore the sulcal pits in children with below‐normal intelligence. Finally, in the present study, ADHD patients showed more asymmetric regions of sulcal pit depth than normal controls, but no significant between‐group differences were detected for the AI of pit depth. It would be helpful to verify this finding with a larger sample in future research.

## 5 CONCLUSION

Taken together, we conducted the first sulcal pits‐based analyses to reveal cortical morphological abnormalities in boys with ADHD. The results confirmed a distinct pattern of pits number between superficial sulci and deep sulci in ADHD relative to controls. Furthermore, significant between‐group differences of pit depth in several regional clusters were observed. These findings help to increase our understanding of ADHD pathology and support the feasibility of sulcal pits as a potential biomarker in the diagnosis of ADHD.

## CONFLICT OF INTEREST

The authors declare no conflict of interest.

## Supporting information

App S1
