# Supplementary material for: Brain morphometric abnormalities in boys with attention‐deficit/hyperactivity disorder revealed by sulcal pits‐based analyses
Source: CNS Neurosci Ther. 2020 Aug 6;27(3):299–307. doi: 10.1111/cns.13445 (PMC7871795; doi:10.1111/cns.13445)

**Table S1. The IDs of ADHD subjects in this study.**

| 10016 | 10017 | 10018 | 10022 | 10025 | 10026 | 10027 | 10028 |
| --- | --- | --- | --- | --- | --- | --- | --- |
| 10030 | 10032 | 10033 | 10035 | 10037 | 10040 | 10042 | 10047 |
| 10049 | 10060 | 10072 | 10091 | 10103 | 10104 | 10106 | 10118 |
| 10119 | 10126 | 10127 | 20008 | 20010 | 20017 | 21002 | 21006 |
| 21015 | 21023 | 21025 | 21026 | 21035 | 21037 | 21039 | 21040 |
| 21042 | 21044 | 21046 | 23002 | 23004 | 23007 | 1019436 | 1023964 |
| 1084283 | 1094669 | 1108916 | 1133221 | 1186237 | 1187766 | 1201251 | 1206380 |
| 1240299 | 1282248 | 1283494 | 1341865 | 1356553 | 1399863 | 1411223 | 1471736 |
| 1511464 | 1517240 | 1577042 | 1643780 | 1743472 | 1780174 | 1784368 | 1809715 |
| 1918630 | 1947991 | 1992284 | 1996183 | 2014113 | 2024999 | 2030383 | 2031422 |
| 2054310 | 2054438 | 2107638 | 2141250 | 2174595 | 2196753 | 2207418 | 2228148 |
| 2230510 | 2260910 | 2276801 | 2288903 | 2292940 | 2299519 | 2367157 | 2380967 |
| 2415970 | 2455205 | 2497695 | 2529026 | 2559559 | 2561174 | 2570769 | 2571197 |
| 2599965 | 2601519 | 2620872 | 2682736 | 2741068 | 2780647 | 2821683 | 2884672 |
| 2910270 | 2919220 | 2940712 | 2950672 | 2950754 | 2996531 | 3052540 | 3124419 |
| 3154996 | 3160561 | 3169448 | 3174224 | 3194757 | 3378296 | 3390312 | 3407871 |
| 3433846 | 3466651 | 3470141 | 3504058 | 3520880 | 3561920 | 3619797 | 3652932 |
| 3653737 | 3672300 | 3677724 | 3679455 | 3691107 | 3712305 | 3767334 | 3803759 |
| 3834703 | 3856956 | 3870624 | 3910672 | 3917422 | 3976121 | 3983607 | 4006710 |
| 4016887 | 4028266 | 4053388 | 4055710 | 4060823 | 4073815 | 4075719 | 4095229 |
| 4095748 | 4154672 | 4187857 | 4221029 | 4225073 | 4241194 | 5302451 | 5993008 |
| 6383713 | 6953386 | 7253183 | 7333005 | 7591533 | 7689953 | 8278680 | 8337695 |
| 8697774 | 8915162 | 9190596 | 9210521 | 9326955 | 9744150 | 9907452 |  |

**Table S2. The IDs of TDC subjects in this study.**

| 10009 | 10010 | 10023 | 10024 | 10039 | 10045 | 10070 | 10082 |
| --- | --- | --- | --- | --- | --- | --- | --- |
| 10117 | 10125 | 10128 | 20001 | 20014 | 20015 | 20016 | 20022 |
| 21005 | 21007 | 21018 | 21034 | 21038 | 23001 | 23005 | 23008 |
| 23011 | 23013 | 23020 | 23027 | 23030 | 23031 | 23035 | 23037 |
| 23038 | 23039 | 1000804 | 1043241 | 1050345 | 1050975 | 1056121 | 1093743 |
| 1117299 | 1132854 | 1253411 | 1320247 | 1359325 | 1386056 | 1404738 | 1408093 |
| 1411536 | 1418396 | 1421489 | 1435954 | 1481430 | 1494102 | 1517058 | 1548937 |
| 1562298 | 1581470 | 1594156 | 1662160 | 1679142 | 1779922 | 1794770 | 1842819 |
| 1849382 | 1860323 | 1875013 | 1879542 | 1884448 | 1988015 | 1995121 | 2018106 |
| 2033178 | 2136051 | 2140063 | 2208591 | 2232413 | 2266806 | 2275786 | 2296326 |
| 2310449 | 2360428 | 2377207 | 2409220 | 2411995 | 2443191 | 2493190 | 2498847 |
| 2511886 | 2528407 | 2554127 | 255899c9 | 2559537 | 2572285 | 2591713 | 2601925 |
| 2618929 | 2640795 | 2703289 | 2735617 | 2740232 | 2833684 | 2917777 | 2920716 |
| 2930625 | 2947936 | 2991307 | 3004580 | 3051944 | 3086074 | 3103809 | 3157406 |
| 3162671 | 3163200 | 3212536 | 3224401 | 3269608 | 3277313 | 3302025 | 3308331 |
| 3320367 | 3349423 | 3385520 | 3473830 | 3494778 | 3562883 | 3593327 | 3610134 |
| 3699991 | 3707771 | 3845761 | 3869075 | 3884955 | 3889095 | 3902469 | 3972956 |
| 3993793 | 3994098 | 4079254 | 4104523 | 4136226 | 4164316 | 4265987 | 4921428 |
| 5216908 | 5575344 | 5669389 | 6346605 | 6477085 | 6550938 | 6592761 | 7093319 |
| 7129258 | 7407032 | 7415617 | 8083695 | 8191384 | 8218392 | 8263351 | 8658218 |
| 8692452 | 8834383 | 9221927 | 9499804 | 9750701 | 9887336 | 9922944 |  |

**Table S3.** **The number of pits in the primary sulci, secondary sulci, dimples and the total brain.**

|  | ADHD  (n = 183) | TDC  (n = 167) | p-value |
| --- | --- | --- | --- |
| Primary sulci | 119.7$\pm$ 7.6 | 120.5 $\pm$ 8.9 | 0.34 |
| Secondary sulci | 75.4 $\pm$ 6.6 | 77.3 $\pm$ 7.0 | 0.01 |
| Dimples | 6.8 $\pm$ 2.1 | 6.6 $\pm$ 2.1 | 0.37 |
| Total brain | 203.0$\pm$ 12.3 | 206.1 $\pm$ 11.5 | 0.02 |

ADHD: attention-deﬁcit/hyperactivity disorder; TDC: typically developing children.

**Table S4. Regions showing significant between-group differences in sulcal pit depth.**

|  | ADHD  (n = 183) | TDC  (n = 167) | p-value |
| --- | --- | --- | --- |
| SFJ.L | 1.59 $\pm$ 0.12 | 1.55 $\pm$ 0.08 | 8.56e-4 |
| CIS.a.L | 1.33 $\pm$ 0.17 | 1.27 $\pm$ 0.13 | 1.95e-4 |
| CIS.b.L | 1.51 $\pm$ 0.09 | 1.47 $\pm$ 0.08 | 9.41e-6 |
| CS.a.L | 1.58 $\pm$ 0.10 | 1.55 $\pm$ 0.07 | 9.80e-4 |
| OS.a.L | 1.22 $\pm$ 0.20 | 1.29 $\pm$ 0.16 | 4.90e-4 |
| IFJ.R | 1.60 $\pm$ 0.10 | 1.57 $\pm$ 0.08 | 7.27e-4 |
| CS.b.R | 1.33 $\pm$ 0.16 | 1.27 $\pm$ 0.10 | 1.54e-4 |
| OS.b.R | 0.66 $\pm$ 0.51 | 0.98 $\pm$ 0.27 | 2.73e-5 |

ADHD: attention-deﬁcit/hyperactivity disorder; TDC: typically developing children; SFJ: superior frontal junction; CIS: circular insular sulcus; CS: cingulate sulcus; OS: orbital sulcus; IFJ: inferior frontal junction; a and b represent different regions of a sulcus. L: left; R: right.

## Appendix S1. Pits Number Analyses using a different division of deep/superficial clusters.

We also classified the cortical folds based on depth, as in Brun et. al^1^. The average DPF of all subjects for each cluster was first calculated, and then a threshold was defined to separate the deep folds from the shallow one. The DPF threshold was selected among the set {1.1, 1.15, 1.2, 1.25, 1.3, 1.35, 1.4} and set to 1.2 in this study so that the deep regions visually contain all primary sulcal folds (e.g. Dubois et al. ^2^, see Figure S1).

**References**

1. Brun L, Auzias G, Viellard M, et al. Localized misfolding within Broca’s area as a distinctive feature of autistic disorder. *Biological Psychiatry: Cognitive Neuroscience and Neuroimaging.* 2016;1(2):160-168.

2. Dubois J, Benders M, Borradori-Tolsa C, et al. Primary cortical folding in the human newborn: an early marker of later functional development. *Brain.* 2008;131(8):2028-2041.

**Figure S1.** **Results for the analyses of pits number****.** (A) Division of cortical folds based on depth. Deep folds with DPF > 1.2 are shown in blue, and shallow folds with DPF ≤ 1.2 are shown in white. (B) Bar charts depicting group differences of the pits number in the deep, shallow and total cortical folds. L: left; R: right; TDC: typically developing children; ADHD: attention-deﬁcit/hyperactivity disorder; DPF, depth potential function; **p* < 0.05.


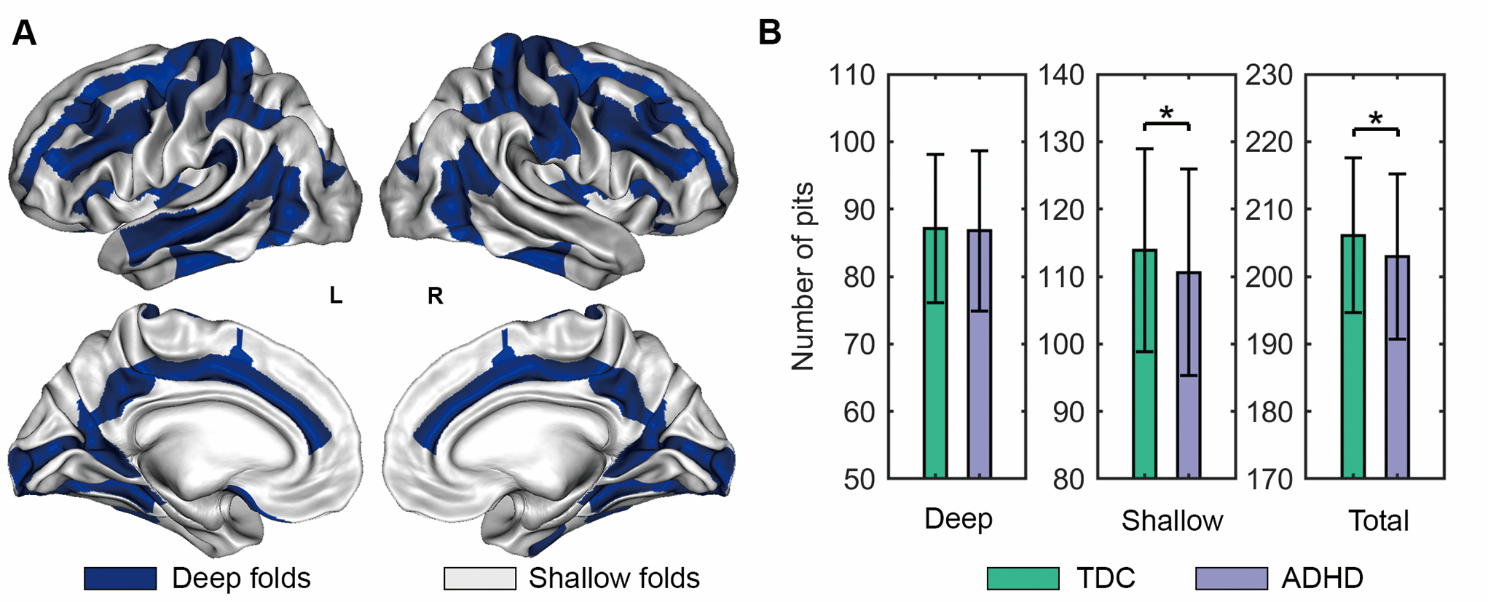

Supplement: Supplementary file 1 — App S1 [file CNS-27-299-s001.docx]
